# Thermal Imaging Metrology Using High Dynamic Range Near-Infrared Photovoltaic-Mode Camera

**DOI:** 10.3390/s21186151

**Published:** 2021-09-13

**Authors:** Thomas B. O. Rockett, Nicholas A. Boone, Robert D. Richards, Jon R. Willmott

**Affiliations:** Department of Electronic and Electrical Engineering, University of Sheffield, Sheffield S10 2TN, UK; t.b.rockett@sheffield.ac.uk (T.B.O.R.); nick.boone@sheffield.ac.uk (N.A.B.); r.richards@sheffield.ac.uk (R.D.R.)

**Keywords:** thermal imaging, radiation thermometry, metrology, infrared, radiometry, photovoltaic camera, log camera

## Abstract

The measurement of a wide temperature range in a scene requires hardware capable of high dynamic range imaging. We describe a novel near-infrared thermal imaging system operating at a wavelength of 940 nm based on a commercial photovoltaic mode high dynamic range camera and analyse its measurement uncertainty. The system is capable of measuring over an unprecedently wide temperature range; however, this comes at the cost of a reduced temperature resolution and increased uncertainty compared to a conventional CMOS camera operating in photodetective mode. Despite this, the photovoltaic mode thermal camera has an acceptable level of uncertainty for most thermal imaging applications with an NETD of 4–12 °C and a combined measurement uncertainty of approximately 1% K if a low pixel clock is used. We discuss the various sources of uncertainty and how they might be minimised to further improve the performance of the thermal camera. The thermal camera is a good choice for imaging low frame rate applications that have a wide inter-scene temperature range.

## 1. Introduction

Photovoltaic (PV) mode cameras have a logarithmic relationship between the incident light intensity and the output camera signal which results in an extremely large dynamic range compared to conventional photodetective (PD) cameras. A large dynamic range is beneficial for thermal imaging applications since the integrated spectral radiance in the near-infrared (NIR) spectral range (0.75–1.1 μm) emitted from a hot target is roughly exponentially proportional to the target temperature in the range 550–3000 °C [1]. Thermal imaging using NIR wavelengths benefits from high performance silicon-based cameras and a lower effect of emissivity uncertainty or variation compared to longer wavelength sensors [2,3,4].

Thermal imaging systems can be evaluated based on their temperature resolution and temperature measurement range. However, there is a trade-off between these two parameters in a conventional linear response PD camera since the thermal radiation intensity is quantified in discrete steps that are unevenly distributed in temperature by the analog to digital converter (ADC) of the camera sensor. The lower temperatures are typically poorly resolved, with adjacent ADC levels corresponding to relatively widely separated temperatures. The opposite is true at higher temperatures, and the ADC levels can be so closely spaced in temperature that they are effectively wasted since their separation is far below the measurement uncertainty. The result is that an NIR thermal imaging system based on a conventional PD sensor can have a good temperature resolution but a poor temperature measurement range as the sensor becomes saturated relatively quickly.

The logarithmic response of a PV camera should, in theory, partially cancel out the exponential increase in radiance emitted from a hot target as a function of temperature. This means the relationship between the camera output signal and target temperature becomes closer to a linear function, and a thermal imaging system based on such a camera will have an enormous temperature measurement range with a roughly constant temperature resolution across this range. This is attractive for thermal imaging applications which have a large temperature variation in the scene.

One application that could benefit from PV thermal imaging is additive manufacturing, in which the peak melt pool temperature can exceed 2500 °C [5] while the surrounding powder is at ambient temperature. The cooling rate of the metal affects its microstructure and hence the bulk properties, meaning that having a high temperature measurement range to image the entire heating and cooling cycle of the part is critically important. Another application is TIG welding [6] where a wide range of temperatures must be sensed in the substrate, without the sensor becoming saturated by the plasma. There are countless other potential applications in high temperature manufacturing and the foundational industries. We note that stacking images with different exposure times can be used to increase the dynamic range, but this is sensitive to movement in the scene [7,8].

Log-response camera sensors have previously been described in the literature for applications with extremely large contrast such as human face analysis in the presence of bright lights [9] and automotive vision applications [10]. Implementations of this technology based on operating the pixels in reverse bias with logarithmic external amplification of the photocurrent, rather than in PV mode, suffered from fixed pattern noise compensation and low sensitivity [11,12,13,14,15].

Log-response ratio thermal imaging has previously been studied [16,17] with a calculated NETD of 5 K and a temperature measurement range of 600–3000 °C. The authors claim an error of 1.5% with a 10-pixel average when imaging a hot filament bulb, though it is not clear how the true temperature of the bulb was measured, or if a blackbody furnace was used. The authors also assumed that ratio thermometry eliminates the need to know the emissivity of the target, which is true only for grey-body targets. In most cases ratio thermometry is less accurate than estimating the measurand emissivity and using a single wavelength thermometer [18].

Understanding how the modified temperature resolution and range of a PV thermal camera impact its measurement uncertainty relative to a conventional PD camera is vitally important to determine which applications could benefit the most from PV thermal imaging. In this work, we quantitively assess the temperature resolution and the temperature measurement range of an uncooled PV mode camera (Magic 1003-1VB, manufactured by NiT) for NIR thermal imaging. The advantages and limitations of NIR thermal imaging with a PV camera are determined by comparison with a conventional thermoelectrically cooled PD camera (Orca Flash 4.0 v3, manufactured by Hamamatsu). The Orca Flash was chosen for comparison as a high-performance cooled CMOS camera that has previously been applied to state-of-the-art NIR thermal imaging [2,6]. The Orca Flash also has a similar pixel size to the Magic camera (6.5 and 6.8 μm, respectively), enabling a comparison of the relevant thermal imaging metrics with only a small difference in the pixel area and the radiance received by each pixel.

The main contribution of this work is to show that, when properly calibrated, a PV thermal camera can achieve an acceptable level of uncertainty and is therefore suitable for many thermal imaging applications. We also show that shifting the “knee” of the logarithmic response of the PV camera to higher temperature by using ND filters provides an insignificant improvement to the temperature resolution at higher temperatures, and therefore establish that a PV camera should be operated with an optically fast lens to improve the low temperature performance of the thermal camera. The results are also applicable to general PV and PD cameras with some extrapolation required based on the quantum efficiency, pixel size, and dynamic range of whichever other camera is considered.

The structure of this paper is described in the following paragraph. The operating principle of the PV camera and relevant thermal imaging metrology metrics are discussed in the methodology section. The results section quantifies the temperature residuals, resolution, noise equivalent temperature difference, fixed pattern noise, and combined measurement uncertainty of the PV thermal camera. Future work and potential optimisations of PV thermal imaging are examined in the discussion section.

## 2. Methodology and Background

The relationship between blackbody radiance *R* and the temperature T of an object with emissivity ε can be described using the semi-empirical Sakuma-Hattori equation [19], see Equation (1). Here A, B, and C are fitting parameters, and $c_{2}$ is the second radiation constant.

(1) $$R\left(T\right)=\frac{\varepsilon C}{\exp\left(\frac{c_{2}}{AT+B}\right)-1}$$

For a traditional CCD or CMOS camera the pixels are operated in reverse bias and have a linear response to radiance, hence the photodetector signal is directly proportional to the blackbody radiance $R\left(T\right)$ and the Sakuma-Hattori equation can be used to calibrate the camera; in effect, converting it from a conventional camera and into a thermal imaging camera.

In PV mode the camera pixels are operated in forward bias and the camera signal $S_{PV}$ is instead proportional to the open circuit voltage $V_{OC}$, which can be found using the Shockley diode equation [20] under illumination at the short-circuit condition, see Equation (2).

(2) $$S_{PV}=DV_{OC}=D\frac{nk_{B}T_{diode}}{q}\ln\left(\frac{I_{L}}{I_{0}}+1\right)$$

Here D is a calibration parameter, n is the ideality factor of the diode, $k_{B}$ is Boltzmann’s constant, $T_{diode}$ is the photodiode temperature, q is the electronic charge, and $I_{0}$ is the saturation current of the diode. The photogenerated current $I_{L}$ is linearly proportional to the blackbody radiance, see Equation (3), where E is a fitting parameter which depends upon the sensor external quantum efficiency, the etendue, and the transmission of the optical system.

(3) $$I_{L}=ER\left(T\right)$$

**Figure 1:**
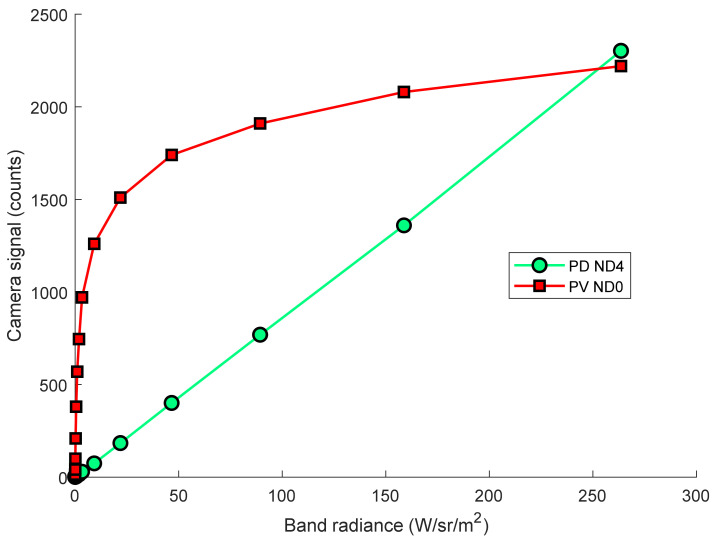
Camera signal of the logarithmic-response PV (with no ND filter) and linear-response PD (with ND4 filter) cameras as a function of the band radiance emitted by the blackbody, including the effect of the 940 nm 10 nm FWHM optical bandpass filter that was included in the optical system.

The signal of the PV camera as a function of temperature can be found by substituting Equations (1) and (3) into (2). The camera signal of the PD and PV cameras used in this work are plotted in Figure 1. As a function of the band radiance emitted by the blackbody.

The strongly sub-linear response of the PV camera is evident in Figure 1. Relevant parameters of the PV camera compared to the PD camera are shown in Table 1.

One critical parameter for a thermal imaging camera is the temperature resolution, which describes how close in temperature two objects can be before they become indistinguishable. The temperature resolution arises since the camera signal is quantified in discrete steps by the ADC. The camera signal is discrete on a more fundamental level since an integer number of incident photons excite (with a probability <1) an integer number of electrons in a pixel. Using a higher bit-depth ADC will not necessarily improve the number of distinguishable temperatures unless the pixel full well capacity is at least as large as the number of ADC levels.

The temperature resolution can be quantified by dT/dS: the change in target temperature per digital level in the camera signal S. Since T(S) is non-linear, the temperature resolution varies with temperature. When measuring lower temperatures, where the signal to noise ratio is reduced, the noise equivalent temperature difference (NETD) is often used to quantify the temperature resolution [21,22]. The *NETD* is defined as the temperature difference between two blackbody targets for which the signal to noise ratio is equal to one and can be experimentally described using Equation (4) [21,23]. Here σ_s_ is the standard deviation in the camera signal at a given target temperature.

(4) NETDT=σsT.dTdS

Another critical parameter in thermal imaging is the dynamic range of the camera sensor. The dynamic range influences the minimum and maximum temperatures that can be accurately measured for a given exposure time and optical train and is dependent on the full well capacity of a camera pixel, the bit depth of the camera’s ADC, the measurement wavelength, and the noise performance of the camera. The dynamic range is defined as the ratio between the highest and lowest signal intensities that the camera is sensitive to (e.g., 37,000:1 or 91 dB): with the highest intensity being the signal at full well capacity, and the lowest signal presumably related to the noise equivalent power (the signal at which the signal to noise ratio equals one with 0.5 s of averaging) [24].

There is a trade-off between temperature resolution and dynamic range, since the camera has a finite number of discrete signal levels which must be spread over a given radiance range: a thermal imaging camera can be sensitive to a wide range of temperatures but have a low temperature resolution, and vice versa. Using the PV mode camera for thermal imaging can result in the temperature resolution being approximately equal at both high and low temperatures, in contrast to a PD camera for which the ADC levels may be unnecessarily closely spaced at higher temperatures, resulting in a temperature resolution which is far below the measurement uncertainty. In practice, whether a thermal imaging camera is optimised for dynamic range or temperature resolution depends on the temperature range in the desired application and the required measurement accuracy.

The thermal imaging cameras studied in this work will also be compared based on how well the experimental calibration points correspond to the physical model of how the camera signal should depend on the target temperature. The uncorrelated components $u_{i}$ that make up the overall standard uncertainty $u_{c}$ can be combined via quadrature addition [25], see Equation (5).

(5) $$u_{c}^{2}=\underset{i=1}{\sum }u_{i}^{2}$$

For reference, the uncertainty of commercial NIR thermal imaging cameras is typically 0.5–2%K: for example, the Land Infrared NIR-2K (0.5–1%K), Optris PI 1M (2%K), and Infratec PIR uc SWIR HD 800 (1%).

**Figure 2:**
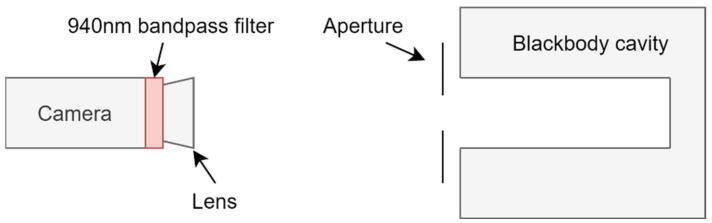
Experimental set-up, showing the camera mounted in front of the blackbody cavity with the 0.6 mm metal aperture in between.

In this work, the pixel values of the two cameras were measured as a function of temperature by sighting them in front of a Land R1500T approximate blackbody cavity, see Figure 2.

The cavity temperature was monitored using a Land Cyclops 100 L single point radiation thermometer that was previously calibrated to UK national standards with an accuracy of ±0.25%K. The cameras were aligned perpendicular to the rear wall of the blackbody cavity and calibrated sequentially to prevent having to realign the cameras for each calibration temperature. This ensured that the cameras were not imaging the cooler side walls of the cavity during their respective calibrations.

Identical optical systems were used for both cameras, except for a 5 mm C-mount extension tube for the CS-mount Magic camera, to achieve the same back focus. A 940 nm (10 nm FWHM) bandpass filter was used to filter the light and a Thorlabs 100 mm f/2.8 lens was used for imaging. Neutral density filters (ND0 to ND4) were placed in front of the camera lens to attenuate the light and set the dynamic ranges of the cameras to simulate using a shorter exposure time.

**Figure 3:**
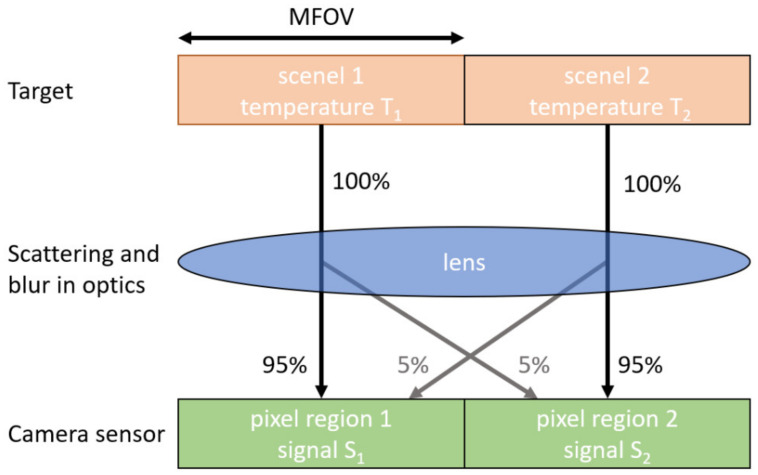
Model for assessing how much temperature measurement uncertainty is caused by photons from a given scenel, of a similar size to the MFOV, being scattered by defects in the optics into nearby areas on the camera sensor.

The measurement field-of-view (MFOV) of the optics, defined as the target diameter from which 95% of the radiance originates [26], was determined to be 0.3 mm at a target distance of 530 mm, using a set of different sized apertures [27]. This was significantly larger than the instantaneous field-of-view (29 μm per pixel using the PD camera). A 0.6 mm aperture was used during the thermal calibration, to ensure that the MFOV was uniformly illuminated while minimising glare. The suitability of this definition of the MFOV was tested by constructing a simple model of two adjacent “scenels” corresponding to the MFOV on the target, see Figure 3.

The extended dynamic range of the PV camera is only useful in practice if objects in the scene do not, due to light scattering and blur in the optics, affect the temperature measurements for adjacent areas. In Figure 3, 95% of the radiance from scenel 1 reaches the corresponding area on the sensor (pixel region 1, which may consist of several pixels), and 5% is scattered to pixel region 2. If scenel 2 happens to be at a much higher temperature than scenel 1, the 5% radiance from scenel 2 that is scattered into pixel region 1 may become significant relative to the 95% radiance originating from scenel 1, causing a large temperature error in pixel region 1. The total radiance received by pixel region 1 is given by Equation (6).

(6) $$R_{1}=0.95R\left(T_{1}\right)+0.05R\left(T_{2}\right)$$

where $R\left(T\right)$ is the radiance emitted by the scenel at temperature T, and is represented by Equation (1). The camera signal measured by pixel region 1 for the PV camera is then found by substituting Equation (6) into Equations (2) and (3), and the temperature error caused by the adjacent hot/cold scenel can be found by comparison with the true temperature of scenel 1.

Dark measurements were taken for every calibration temperature and subtracted from the measured camera signal. The ND2 filter used in this work had an optical density of 1.753 at the measurement wavelength of 940 nm (transmission = 0.55%), measured using the PD camera.

## 3. Results

### 3.1. Thermal Calibration

**Figure 4:**
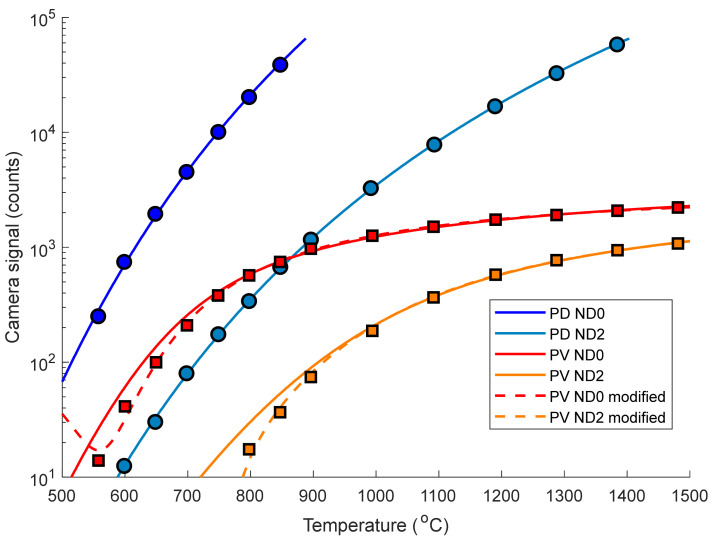
Camera signal as a function of temperature for the photodetective and photovoltaic cameras (markers). Data with and without an ND2 filter are shown here, with the measurements made using other ND filters omitted for clarity. The lines represent fits: the PV camera data are simulated using Equation (2) (solid lines) and the modified model in Equation (7) (dashed lines). The PD camera data are simulated using Equation (1) (solid lines).

The camera signal with the dark offset subtracted at each target temperature is plotted in Figure 4, alongside a corresponding fit to the data using Equation (1) for the PD camera and Equation (4) for the PV camera. The model for how the PV camera signal should vary with temperature conformed poorly to the experimental data particularly below 800 °C, so Equation (2) was modified with a temperature dependent adjustment, see Equation (7).

(7) $$S_{PV\;modified}=S_{PV}+F\left(T-G\right)$$

where *F* and *G* are fitting parameters. This modified model produced a closer fit to the experimental data but has no physical basis. The model in Equation (7) is poorly constrained outside the calibration range (550–1500 °C) so this model cannot be used to extrapolate temperatures above 1500 °C, whereas the model given in Equation (2) can (with an increasing uncertainty the higher the temperature).

The limited dynamic range of the PD camera is evident as it reaches saturation (65,535 counts) at 890 °C whereas, using Equation (2), the PV camera should reach saturation at around one billion kelvins. This highlights the immense dynamic range of the PV camera. Attenuating the radiance using an ND2 filter modified the measurement range of the PD camera to 670–1400 °C, enabling a comparison with the PV camera over a wider temperature range.

### 3.2. Deviation of Model from Experimental Data

**Figure 5:**
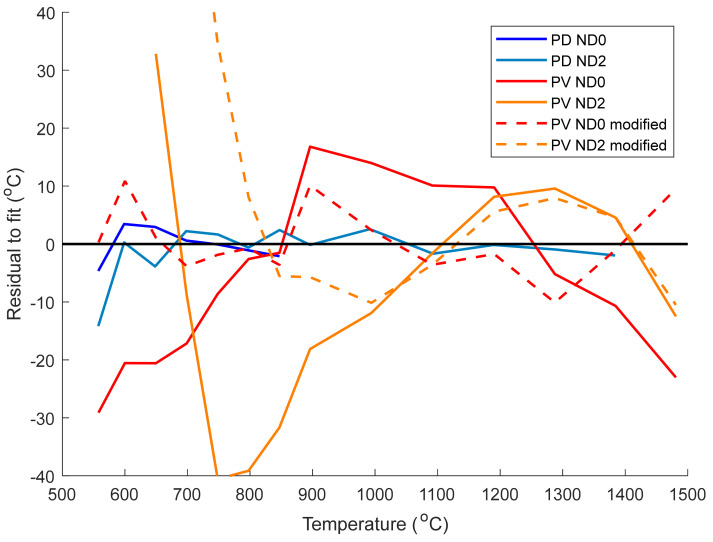
Temperature residuals of the fitting models plotted in Figure 4 as a function of temperature. Data with and without an ND2 filter are shown, with the measurements made using other ND filters omitted for clarity. Temperature residuals for the PV camera were obtained using Equation (2) (solid lines) and the modified model in Equation (7) (dashed lines).

The uncertainty caused by the imperfect fitting of the model to the calibration data was quantified using the temperature residual at each data point, see Figure 5. If the modelled fit and the data aligned closely then a line close to zero with randomly residual values would be expected. The residuals of the PV camera (solid red and orange lines) are not randomly distributed, indicating that Equation (2) is not sufficient to fit the data accurately. The modified model of the PV camera signal (dotted red and orange lines) appears to have randomly distributed residuals and fits the data more closely than Equation (2). The PV camera with the ND2 filter (orange dashed line) deviates markedly from the model below around 900 °C as the camera signal approaches the noise floor.

From Figure 5, the mean absolute temperature residuals of the PV camera are 13.5 and 17.8 °C with the ND0 and ND2 filters, respectively, while using the modified model in Equation (7) results a large reduction (values of 4.3 with ND0 and 9.6 °C with ND2). For reference, the mean absolute temperature residuals of the PD camera are 2.1 and 2.5 °C with the ND0 and ND2 filters, respectively. The temperature residuals using the PD camera are much lower than the PV camera even when using the modified model in Equation (7), highlighting the difficulty in calibrating the PV camera for thermal imaging.

### 3.3. Temperature Resolution

**Figure 6:**
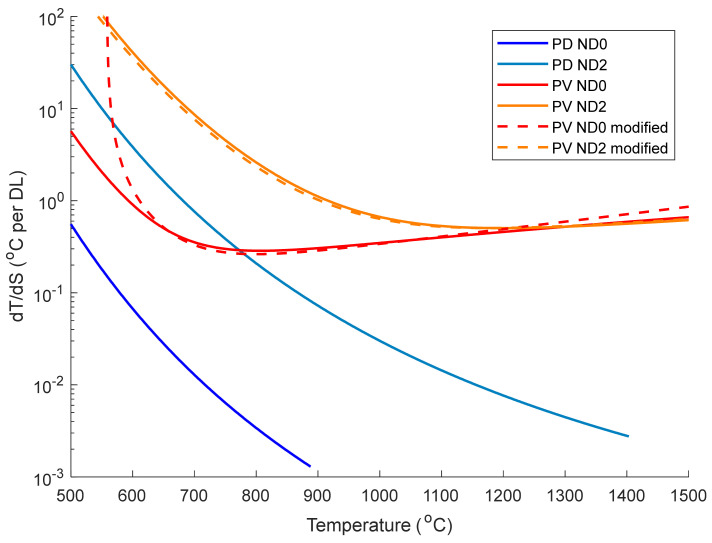
Temperature resolution dT/dS of the photodetective and photovoltaic cameras as a function of temperature. Data with and without an ND2 filter are shown here, with the measurements made using other ND filters omitted for clarity. The temperature resolution of the PV camera was calculated using Equation (2) (solid lines) and the modified model in Equation (7) (dashed lines).

The temperature resolution dT/dS of the two cameras in terms of °C change per digital level was calculated by differentiating the fitting models (Equations (1), (2) and (7)) and is plotted in Figure 6.

One would expect the temperature resolution to decrease exponentially with temperature due to the exponential increase in the radiance with temperature: this is the case for the PD camera in Figure 6. However, the PV camera has a relatively constant temperature resolution across the entire temperature range due to its logarithmic response to radiance cancelling out the exponential increase in radiance as the temperature increases.

The temperature resolution of the PV camera with an ND2 filter becomes superior to the unfiltered ND0 case above 1300 °C (characterised by the red and orange lines crossing over in Figure 6) due to the ND2 filter shifting the “knee” of the logarithmic response seen in Figure 4 to higher temperature. However, this improvement is miniscule in this temperature range and is not worth the loss in signal and corresponding increase in minimum resolvable temperature. This suggests that using minimal ND filtering and a faster lens would improve the low temperature noise performance without drastically affecting the high temperature resolution.

The PV camera has a far worse temperature resolution than the PD camera (comparing the dark blue and red lines). This is due to the lower bit depth of the PV camera pixel readout electronics (14 vs. 16 bit), the PV camera not using the full capacity of its ADC (the signal only increases by ~2000 counts from 550–1500 °C), and the flattened logarithmic response of the camera (especially above the “knee” point at around 800 °C). However, the temperature resolution of the PV camera is around 0.4 °C over most of the temperature range, which is adequate for many applications.

### 3.4. NETD

**Figure 7:**
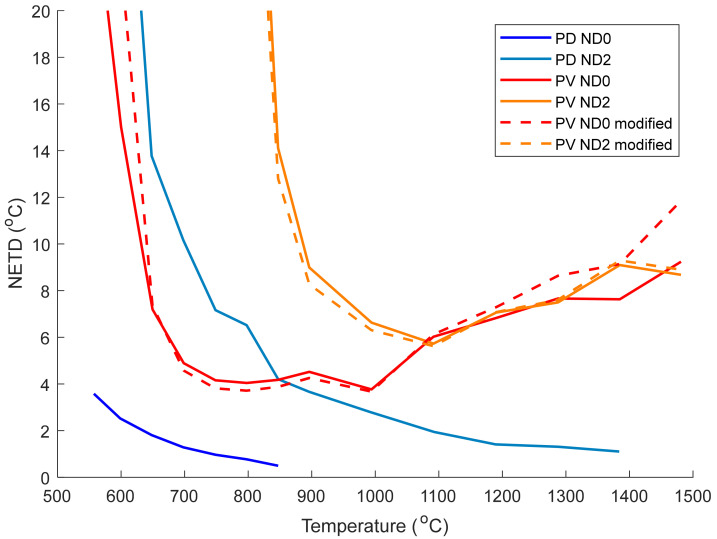
NETD of the photodetective and photovoltaic cameras as a function of temperature. Data with and without an ND2 filter are shown here, with the measurements made using other ND filters omitted for clarity. The NETD of the PV camera was calculated using both Equation (2) (solid lines) and Equation (7) (dashed lines).

The NETD of the two cameras was calculated using Equation (4), using the standard deviation of a single pixel in the calibration images (100 frames at each temperature), see Figure 7. The value of σ_s_ for the PD camera followed a square root dependence on the signal indicating that the camera was shot noise limited (not shown here). However, the PV camera had a nearly constant value of σ_s_ meaning that noise in the readout electronics or thermal noise in the sensor is the dominant source of noise.

The NETD of the PV camera without the ND filter is 4–12 °C in the temperature range 630–1500 °C, similar to the value of 5 K reported in [17]. This is significantly worse than the 0.5–3.5 °C NETD of the PD camera. However, if a thermal imaging camera with measurement range 630–1500 °C were desired, an interesting comparison is the PV camera (with no ND filter) and the PD camera (with ND2 filter), the red and light blue lines in Figure 7. In this case the two cameras have a similar NETD, with the PV camera performing better in the range 600–850 °C, and the PD camera having a lower NETD in the range 850–1500 °C (a region where the acceptable uncertainty is larger).

### 3.5. Fixed Pattern Noise

**Figure 8:**
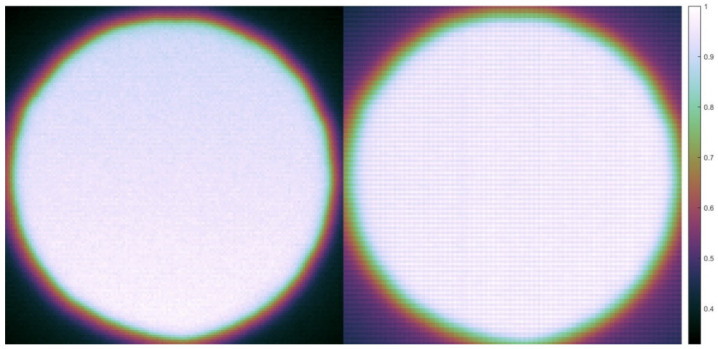
Raw images of blackbody furnace taken using the PV camera viewed through an aperture at different pixel clocks; left 12.5 MHz, right 80 MHz. Fixed pattern noise is visible in the right image. The corners of the right image are also brightened relative to the left, indicating that the modulation transfer function of the imaging system is negatively affected by the higher pixel clock.

Raw images of the blackbody furnace at 1480 °C with pixel clocks of 12.5 and 80 MHz are shown in Figure 8. The fixed pattern noise is clearly visible in the right image (80 MHz pixel clock) and is reduced below the level of the other noise (dark current, read out noise etc.) if the pixel clock is 12.5 MHz. The fixed pattern noise at higher pixel clocks may restrict thermal imaging using the PV camera to lower frame rates and prevent its application to fast processes such as additive manufacturing, although future advances in camera technology may overcome this problem. The increased camera signal outside the aperture with the higher pixel clock (shown by the purple corners of Figure 8 right) is probably due to pixel cross-talk and shows that using a high pixel clock also negatively affects the modulation transfer function of the imaging system.

### 3.6. Combined Measurement Uncertainty

**Figure 9:**
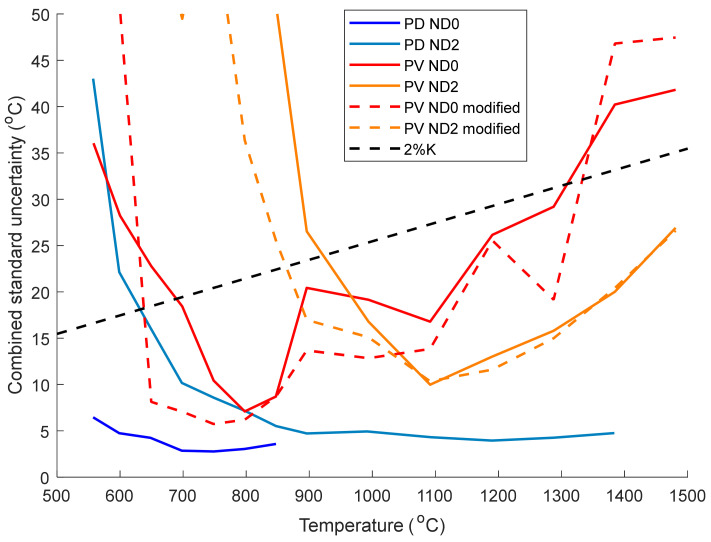
Combined standard uncertainty of the photodetective and photovoltaic cameras as a function of temperature. Data with and without an ND2 filter are shown here, with the measurements made using other ND filters omitted for clarity. The uncertainty of the PV camera was calculated using both Equation (2) (solid lines) and Equation (7) (dashed lines). An uncertainty of 2% K (black dashed line) is also plotted for reference.

The combined measurement uncertainty of the two cameras was calculated using Equation (5) and is displayed in Figure 9 along with a black line corresponding to an uncertainty of 2% K which is typical for commercial thermal imaging cameras. The values in the uncertainty budget are shown in Table 2. Two sources of uncertainty that are difficult to minimise are the furnace temperature uncertainty (due to the 0.25% K uncertainty of the Cyclops 100 L reference thermometer) and the furnace back wall uncertainty (caused by a small non-uniformity in the temperature of the blackbody furnace). The lens did exhibit significant vignetting: a 15% fall off in intensity 6 mm from the centre of the PD camera sensor. However, the calibration points were all taken within 0.5 mm of the centre where the vignetting was only around 0.03% of the signal. The uncertainty due to the fixed pattern noise was approximated as half of the difference between the light and dark lines in the thermal images.

From Figure 8, the PV camera has a larger uncertainty than the PD camera, but it is still close to 2% K above 620 °C without an ND filter (red lines). Given that for real world applications the uncertainty in the target emissivity is likely to be larger than 2%, the PV camera shows an acceptable level of uncertainty and is therefore suitable for scientific and industrial thermal imaging.

**Figure 10:**
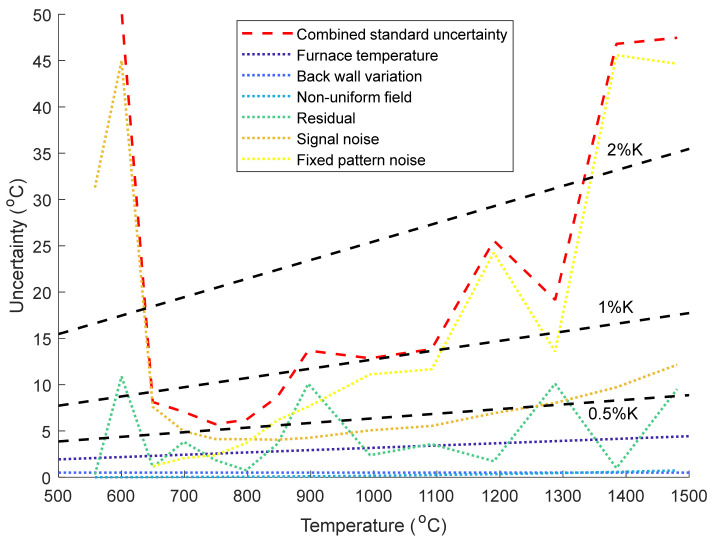
Variation in the sources of uncertainty as a function of temperature (dotted lines) for the PV camera (with no ND filter) using the modified model in Equation (7). Additionally shown are the combined standard uncertainty (red dashed line) and uncertainties of 0.5, 1, and 2% K (black dashed lines) for reference.

To further investigate the origin of the larger uncertainty of the PV camera, each source of uncertainty in Figure 9 is plotted as a function of temperature in Figure 10, for the case with no ND filter and the modified calibration in Equation (7).

From Figure 10, below 650 °C the uncertainty is dominated by noise (a combination of the readout noise and shot noise, this does not include fixed pattern noise). Above 650 °C the fixed pattern noise is the dominant source of uncertainty, although we note that this can be nearly eliminated by using a lower pixel clock at the expense of the frame rate. The other main sources of uncertainty above 650 °C are uncertainty in the furnace temperature, noise in the signal, and poor fitting of the model to the calibration data. The 0.25% K uncertainty in the furnace temperature cannot be improved unless a more accurate reference thermometer were used. The signal noise is a combination of shot noise, the dark current in the silicon, and noise in the electronic readout circuits. The dark current could be reduced by cooling the camera, and the shot noise reduced by using an optically faster lens and/or wider wavelength bandpass for the measurement. The poor fitting of the model to the calibration data requires further study in order to produce an accurate fit. Another area for improvement is real time image conversion: we note that Equation (7) is more computationally intensive than Equation (2). A look-up table formed by combining Equation (7) for the low temperatures <800 °C and Equation (2) for the high temperatures >800 °C would be computationally faster than solving Equation (7) in real time, and allow the extrapolation of temperatures outside the calibration range >1500 °C.

### 3.7. Test Application Images

**Figure 11:**
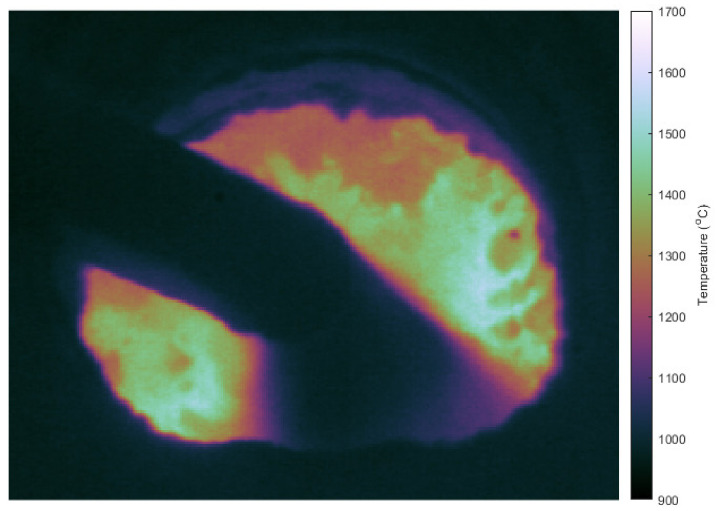
Example thermal image from the PV camera looking into through a window into a blast furnace. The tuyere and the injected coal powder, which are at temperatures below the detection limit of the camera, are visible as the black objects in the middle of the image. The fixed pattern noise has been removed by binning the image, at the expense of resolution.

Once the PV camera metrology study was complete, the efficacy for real-world applications requiring high dynamic range was undertaken. Test thermal images were taken sighting the PV camera at a blast furnace coal injection Tuyere (TATA Steel, Port Talbot, Wales), see Figure 11 for a representative example image from the thermal video stream.

Initial temperature measurements of the tuyere with the PV camera were around 500 °C lower than values measured concurrently with a PD camera. The ambient temperature in the furnace room was estimated to be 40–50 °C during the test, compared to 25 °C during the lab calibration. The dark offset of the sensor was measured as a function of the ambient temperature using an environmental chamber and found to decrease linearly by −18.6 °C^−1^. The ambient temperature also affects $V_{OC}$ of the camera sensor, see Equation (2). These two factors combine to cause the temperature measurement to be greatly underestimated at high ambient temperatures. A correction was applied to Figure 11 to compensate for this. This highlights the extreme sensitivity of the PV thermal imaging camera to ambient temperature and means that sensor temperature stabilisation or cooling and in situ dark frame measurements are necessary for operation outside of a climate-controlled laboratory.

### 3.8. Temperature Error from Size of Source Effect

**Figure 12:**
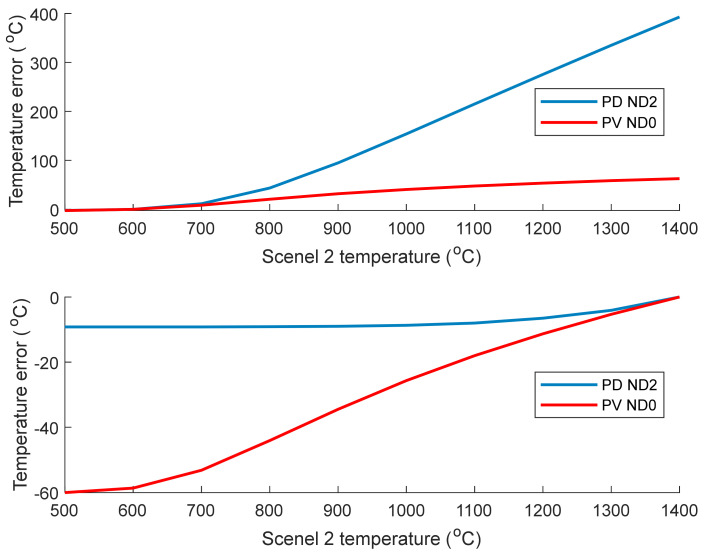
Temperature error in pixel region 1 simulated using the model displayed in Figure 3 when the adjacent scenel 2 is at a different temperature. Top: scenel 1 temperature 600 °C. Bottom: scenel 1 temperature 1400 °C. Note that when both scenels are at the same temperature, the temperature error is zero.

In this section the temperature error caused by the size of source effect when an adjacent hot or cold object is next to the MFOV is considered for the two camera/ND filter combinations that have the most useful temperature measurement range: the PV camera with no ND filter, and the PD camera with an ND2 filter. This test shows how well the thermal imaging systems can accurately resolve the temperature of closely spaced objects that are at different temperatures, see Figure 12.

From Figure 12, the top graph (scenel 1 temperature of 600 °C) shows that the PV camera has a far smaller temperature error than the PD camera when the adjacent scenel is at a higher temperature, with an error of 62 °C compared to 390 °C for the PD camera when scenel 2 is at 1400 °C. This is expected since the signal measured by the PD camera is exponentially dependent on temperature so a small increase in the temperature of scenel 2 leads to a drastic increase in the amount of light that is scattered or blurred into pixel region 1. From the bottom graph in Figure 12, the PD camera has a smaller temperature error than the PV camera when scenel 2 is at a lower temperature. However, the PV camera only has an error of −60 °C in the extreme case when scenel 2 is at 500 °C. These results highlight that the PV camera can resolve the temperature of objects more accurately when there is a large temperature variation over a small part of the image or when there are high temperature gradients in the scene, as in additive manufacturing and welding applications.

## 4. Discussion and Conclusions

The temperature resolution and uncertainty of the PV camera, despite being worse than the PD camera, are acceptable for high temperature thermal imaging applications. The large dynamic range of the camera is ideal for imaging applications that have a large intra-scene temperature range.

Additive manufacturing could benefit from thermal imaging using a PV camera, although we note that the camera used in this work does not have a high enough frame rate to extract meaningful information about the extremely high heating and cooling rates in processes such as selective laser melting and electron beam melting. Furthermore, the fixed pattern noise is the main source of uncertainty unless the pixel clock, and therefore the frame rate, is reduced. Welding and furnace applications could be ideal scenarios for this instrument.

Temperature stabilisation of the sensor is critical for thermal imaging with the PV camera, as shown by the large underestimate of the measured temperature in the hot blast furnace environment.

Operating the camera at a shorter wavelength would result in a marginally improved temperature resolution, at the expense of a higher minimum resolvable temperature. A wider wavelength bandpass and an optically faster lens would increase the amount of light reaching the sensor and lead to less shot noise at lower target temperatures.

To conclude, we report on the measurement uncertainty of a near infrared thermal imaging camera based on a photovoltaic mode camera. The large dynamic range of the camera translated to a superior temperature measurement range, compared to a conventional photodiode mode camera. The main source of temperature uncertainty was fixed pattern noise, which could be alleviated by operating the sensor at a lower pixel clock and frame rate. The sensor is difficult to saturate, therefore the low temperature noise performance could be improved by using a faster lens and/or wider wavelength range for measurement with only a miniscule impact on the high temperature resolution. The temperature resolution and uncertainty of the photovoltaic camera were around 0.4 °C and approximately 2% K, respectively, which is acceptable for many thermal imaging applications.

The major findings of this study were that for relatively low frame rate applications, the PV sensor is a good choice in scenarios where a very wide dynamic range is required; neutral density filtering is always counterproductive because it reduces low temperature performance without improving high temperature performance; the PV sensor is superior to the conventional PD camera at accurately representing high temperature gradients; the measurement uncertainty due to sensor noise can be made to be approximately 1% K across a very wide range of temperatures if a low pixel clock is used.

## Figures and Tables

**Table 1:** Specifications of the PV camera and reference PD camera studied for NIR thermal imaging.

Camera	Magic MC1003-1VB	Orca Flash 4.0 v3
Manufacturer	NiT	Hamamatsu
Resolution	1280 × 1024	2048 × 2048
Pixel size (μm)	6.8	6.5
ADC (bits)	14	16
Dynamic range (dB)	140	91.3
Exposure time used (ms)	30.303	30
Sensor cooling	No	−10 °C
Operating mode	Photovoltaic (forward bias)	Photodetective (reverse bias)

**Table 2:** Components of combined uncertainty.

Uncertainty	Value
Furnace temperature	0.25% K (uncertainty of reference thermometer)
Furnace back wall variation	0.5 K
Residual to fit	See Figure 5
Non-uniform field	0.03% S
Signal noise	Standard deviation of single pixel over 100 frames
Fixed pattern noise	Half of the difference between light and dark lines in thermal image

## Data Availability

Data available in a publicly accessible repository at 10.6084/m9.figshare.16610476.
